# Solitary benign metastasizing leiomyoma: imaging features and pathological findings

**DOI:** 10.1590/S1806-37132014000200015

**Published:** 2014

**Authors:** Bernardo Corrêa de Almeida Teixeira, Kássia Mahfouz, Dante Luiz Escuissato, Ana Flávia Cardoso Buarque Costa, Lúcia de Noronha

**Affiliations:** Federal University of Paraná Hospital de Clínicas, Curitiba, Brazil; Federal University of Paraná, Curitiba, Brazil; Federal University of Paraná, Curitiba, Brazil; Federal University of Paraná Hospital de Clínicas, Curitiba, Brazil; Federal University of Paraná, Curitiba, Brazil

## To the Editor:

A 51-year-old woman presented with dyspnea on exertion, dry cough, dyslipidemia, type 2 diabetes, and liver steatosis. Twenty years prior, she had undergone hysterectomy and unilateral oophorectomy because of uterine leiomyomas.

**Figure 1:**
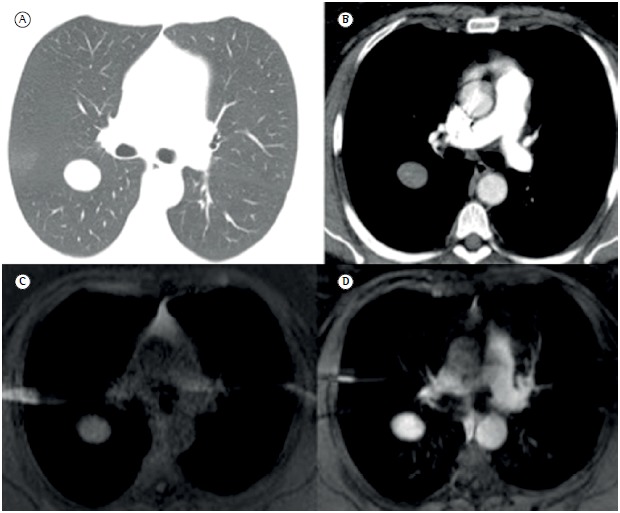
CT scans and magnetic resonance imaging of the chest. In A and B, chest CT scans (lung window, in A, and mediastinal window, in B) showing an oval nodule with homogeneous density, well-defined margins, and contrast enhancement. In C and D, fat-suppressed T1-weighted images (before injection of a paramagnetic contrast agent, in C, and after injection of a paramagnetic contrast agent, in D), on which the nodule is slightly hyperintense and homogeneously enhanced.

A chest X-ray showed a solitary pulmonary nodule in the right upper lobe. The nodule was oval in shape, being approximately 24 mm × 30 mm. A CT scan of the chest confirmed that the lesion was a solitary, well circumscribed-nodule that had regular borders and was located in the posterior segment of the right upper lobe. As can be seen in Figures 1A and 1B, there was delayed contrast enhancement (50 HU before contrast injection, 55 HU within 25 s after contrast injection, and 100 HU within 5 min after contrast injection). Contrast-enhanced magnetic resonance imaging was performed, and the nodule showed slightly high signal intensity on T1-weighted images and homogeneous enhancement (Figures 1C and 1D). The lesion showed signal intensity similar to that of muscle on T2-weighted images. In-phase and out-of-phase imaging, fat-saturated imaging, and diffusion-weighted imaging provided no additional findings. A chest X-ray performed three years earlier had shown no lesions.

**Figure 2:**
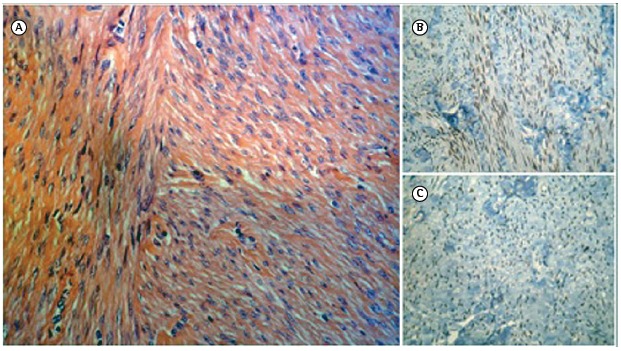
Photomicrographs of the pulmonary nodule. In A, note that the nodule consisted of smooth muscle tissue arranged in multidirectional bundles, without atypia or mitosis (H&E; magnification, ×400). In B, note progesterone receptor positivity (immunohistochemistry; magnification, ×100). In C, note estrogen receptor positivity (immunohistochemistry; magnification, ×100).

Because the imaging findings were inconclusive and because of the risk of malignancy, the patient underwent video-assisted thoracoscopic surgery for nodule resection. Pathological examination revealed a nodular proliferation composed of smooth muscle cells without atypia and areas of hyalinization, a finding that was consistent with leiomyoma (Figure 2A). Immunohistochemical analysis of the lesion showed that estrogen receptors and progesterone receptors were positive, and a diagnosis of benign metastasizing leiomyoma (BML) was made despite the atypical presentation, i.e., a solitary pulmonary nodule (Figures 2B and 2C).

BML is a rare neoplastic process in which leiomyomas of the uterus metastasize to distant sites, the most common of which are the lungs.^(^ ^1^ ^,^ ^2^ ^)^ BML is usually asymptomatic, and the diagnosis is based on incidental imaging findings of multiple pulmonary nodules or, more rarely, a single nodule. The term metastasizing fibroleiomyoma of the uterus was introduced by Steiner in 1939 to described multiple nodules of proliferating smooth muscle cells in the lung of women with a history of hysterectomy.^(^ ^2^ ^)^ Different mechanisms of spread of uterine leiomyomas have been proposed. It has been suggested that smooth muscle cells spread to the lungs after uterine extension into pelvic venous channels; that tumors gain venous access from surgical trauma during hysterectomy; and that the lesions represent metastatic foci arising from low-grade leiomyosarcomas.^(^ ^3^ ^,^ ^4^ ^)^

In cases of BML, pulmonary nodules can be seen 3-240 months after hysterectomy or even before the procedure. They can vary in size from millimeters to centimeters and be randomly distributed in the lung parenchyma.^(^ ^3^ ^,^ ^4^ ^)^ A solitary nodule, as seen in our patient, is a very rare presentation of BML. In general, pulmonary nodules do not calcify and can remain unchanged or even regress spontaneously. Both CT and magnetic resonance imaging can be used in order to characterize pulmonary nodules in patients with BML; such nodules have a nonspecific appearance and usually show homogeneous contrast enhancement.^(^ ^3^ ^,^ ^5^ ^)^ The efficacy of 18F-fluorodeoxyglucose positron emission tomography with CT (FDG-PET/CT) in detecting uterine leiomyomas is controversial. In the few reports available in the literature, FDG-PET/CT was unable to detect BML.^(^ ^6^ ^)^ In the case reported here, FDG-PET/CT was not performed.

Macroscopically, pulmonary nodules are ovoid, well circumscribed, and homogeneously white. Microscopic examination reveals proliferation of well-differentiated, benign-appearing spindle cells with eosinophilic cytoplasm, moderate degree of vascularization, insignificant nuclear atypia, mitotic activity, anaplasia, necrosis, vascular invasion, or inflammatory host tissue response. The presence of estrogen receptors and progesterone receptors in cases of BML has been well documented and constitutes evidence that BML originates from uterine smooth muscle. Extrauterine leiomyomas are uniformly estrogen receptor negative. In contrast, most BMLs are estrogen receptor positive.^(^ ^4^ ^)^ The disease course varies and seems to depend on the estrogen status of the patient. In postmenopausal women, the disease is indolent, patient mortality being commonly due to an unrelated disease process, whereas, in premenopausal women, the progression of the disease can result in death.^(^ ^1^ ^,^ ^3^ ^)^

Because BML is a rare disease, with few reported cases, there is no established treatment protocol. Given that BML is a hormonally responsive tumor, the prognosis is favorable.^(^ ^1^ ^,^ ^3^ ^)^ Treatment includes hysterectomy, bilateral oophorectomy, and long-term hormone therapy. Expectant management and pulmonary nodule resection are also therapeutic options. Menopause has been associated with lesion regression.
