# Supplementary material for: Solitary benign metastasizing leiomyoma: imaging features and pathological findings
Source: J Bras Pneumol. 2014 Mar-Apr;40(2):193–5. doi: 10.1590/S1806-37132014000200015 (PMC4083647; doi:10.1590/S1806-37132014000200015)
Supplement: Translation

# Translation: Leiomioma metastático benigno solitário: aspectos de imagem e achados anatomopatológicos

**Authors:** Bernardo Corrêa de Almeida Teixeira, Kássia Mahfouz, Dante Luiz Escuissato, Ana Flávia Cardoso Buarque Costa, Lúcia de Noronha

**Affiliations:** Hospital de Clínicas da Universidade Federal do Paraná, Curitiba (PR) Brasil; Universidade Federal do Paraná, Curitiba (PR) Brasil; Universidade Federal do Paraná, Curitiba (PR) Brasil; Hospital de Clínicas da Universidade Federal do Paraná, Curitiba (PR) Brasil; Universidade Federal do Paraná, Curitiba (PR) Brasil

## Ao Editor:

Uma mulher de 51 anos de idade apresentou dispneia aos esforços, tosse seca, dislipidemia, diabetes tipo 2 e esteatose hepática. Vinte anos antes, a paciente havia sido submetida a histerectomia e ooforectomia unilateral por causa de leiomiomas uterinos.

A radiografia de tórax mostrou um nódulo pulmonar solitário no lobo superior direito. O nódulo era oval e media aproximadamente 24 mm × 30 mm. A TC de tórax confirmou que a lesão era um nódulo solitário bem circunscrito, com margens regulares, localizado no segmento posterior do lobo superior direito. Como se pode observar nas Figuras 1A e 1B, o realce da lesão após a administração do contraste tardou (50 UH antes da injeção do contraste, 55 UH 25 s após a injeção do contraste e 100 UH 5 min após a injeção do contraste). Na ressonância magnética com contraste, o nódulo apresentou sinal de intensidade levemente alto e realce homogêneo nas imagens ponderadas em T1 (Figuras 1C e 1D). A lesão mostrou sinal de intensidade semelhante à intensidade muscular nas imagens ponderadas em T2. As sequências de imagem *in-phase* e *out-of-phase*, com saturação de gordura e ponderada em difusão não propiciaram achados adicionais. A radiografia de tórax realizada três anos antes não revelou nenhuma lesão.

**Figura 1:** TC e ressonância magnética de tórax. Em A e B, TC de tórax (em A, janela para o pulmão; em B, janela para o mediastino) mostrando um nódulo oval com densidade homogênea, margens bem definidas e realce após a administração de contraste. Em C e D, imagens ponderadas em T1 com supressão de gordura (em C, antes da injeção de contraste paramagnético; em D, após a injeção de contraste paramagnético), nas quais o nódulo está ligeiramente hiperintenso e homogeneamente realçado.

Como os achados de imagem foram inconclusivos e havia risco de malignidade, a paciente foi submetida a videotoracoscopia para a resseção do nódulo. O exame anatomopatológico revelou uma proliferação nodular composta por células musculares lisas sem atipia e áreas de hialinização, um achado consistente com leiomioma (Figura 2A). A análise imuno-histoquímica da lesão revelou positividade para receptores de estrógeno e progesterona; fez-se o diagnóstico de leiomioma metastático benigno (LMB), não obstante a apresentação atípica, isto é, um nódulo pulmonar solitário (Figuras 2B e 2C).

**Figura 2:** Fotomicrografias do nódulo pulmonar. Em A, nota-se que o nódulo consiste em tecido muscular liso disposto em feixes multidirecionais, sem atipia ou mitose (H&E; aumento: 400×). Em B, nota-se positividade para receptores de progesterona (imuno-histoquímica; aumento: 100×). Em C, nota-se positividade para receptores de estrógeno (imuno-histoquímica; aumento: 100×).

O LMB é um processo neoplásico raro no qual há metástase de leiomiomas do útero para sítios distantes, mais comumente os pulmões.^(^ ^1^ ^,^ ^2^ ^)^ O LMB é geralmente assintomático, e o diagnóstico baseia-se em achados incidentais de imagem de múltiplos nódulos pulmonares ou, mais raramente, de um único nódulo. O termo fibroleiomioma metastático do útero foi introduzido por Steiner em 1939 para descrever múltiplos nódulos de células musculares lisas que se proliferavam no pulmão de mulheres com história de histerectomia.^(^ ^2^ ^)^ Foram propostos diferentes mecanismos de disseminação de leiomiomas uterinos. Sugeriu-se que as células musculares lisas disseminam-se para os pulmões após a extensão do útero para os canais venosos pélvicos; que tumores ganham acesso venoso a partir de trauma cirúrgico durante a histerectomia e que as lesões representam focos metastáticos provenientes de leiomiossarcomas de baixo grau.^(^ ^3^ ^,^ ^4^ ^)^

Em casos de LMB, nódulos pulmonares podem ser vistos de 3 a 240 meses após a histerectomia ou mesmo antes do procedimento. O tamanho dos nódulos pode variar (de milímetros a centímetros), e eles podem estar aleatoriamente distribuídos no parênquima pulmonar.^(^ ^3^ ^,^ ^4^ ^)^ Um nódulo solitário, como o que foi encontrado em nossa paciente, é uma apresentação muito rara de LMB. Em geral, os nódulos pulmonares não calcificam e podem permanecer inalterados ou até mesmo regredir espontaneamente. Tanto a TC como a ressonância magnética podem ser usadas para caracterizar nódulos pulmonares em pacientes com LMB; tais nódulos têm aparência inespecífica e geralmente apresentam realce homogêneo após a administração de contraste.^(^ ^3^ ^,^ ^5^ ^)^ A eficácia da tomografia por emissão de pósitrons com 18F fluordesoxiglicose (FDG-PET, do inglês *18F-fluorodeoxyglucose positron emission tomography*) com TC (FDG-PET/TC) no que tange à detecção de leiomiomas uterinos é controversa. Nos poucos relatos existentes na literatura, a FDG-PET/TC não detectou LMB.^(^ ^6^ ^)^ No caso aqui relatado, a FDG-PET/TC não foi realizada.

Macroscopicamente, os nódulos pulmonares são ovoides, bem circunscritos e homogeneamente brancos. O exame microscópico revela proliferação de células fusiformes bem diferenciadas e de aparência benigna, com citoplasma eosinofílico e grau moderado de vascularização, atipia nuclear insignificante, atividade mitótica, anaplasia, necrose, invasão vascular ou resposta inflamatória do tecido hospedeiro. A presença de receptores de estrógeno e progesterona em casos de LMB está bem documentada e é prova de que o LMB provém da musculatura lisa uterina. Leiomiomas extrauterinos são uniformemente negativos para receptores de estrógeno. Por outro lado, a maioria dos LMB é positiva para receptores de estrógeno.^(^ ^4^ ^)^ O curso da doença varia e parece depender do estado estrogênico de cada paciente. Em mulheres na pós-menopausa, a doença é indolente (e o óbito comumente se dá em virtude de um processo de doença não relacionado), ao passo que em mulheres na pré-menopausa a progressão da doença pode resultar em óbito.^(^ ^1^ ^,^ ^3^ ^)^

Como o LMB é uma doença rara, com poucos casos relatados, não há nenhum protocolo de tratamento estabelecido. Como o LMB é um tumor que reponde a hormônios, o prognóstico é favorável.^(^ ^1^ ^,^ ^3^ ^)^ O tratamento inclui histerectomia, ooforectomia bilateral e terapia hormonal em longo prazo. A conduta expectante e a resseção dos nódulos pulmonares também são opções terapêuticas. Há uma relação entre a menopausa e a regressão da lesão.
